# Incidental finding of a *DMD* exons 48–55 deletion during prenatal diagnosis

**DOI:** 10.3389/fped.2025.1541468

**Published:** 2025-04-17

**Authors:** Min Zhang, Zhaodong Lin, Meihuan Chen, Danhua Guo, Qiaomei Yang, Qianqian He, Bin Mao, Bin Liang, Lingji Chen, Meiying Cai, Hailong Huang, Liangpu Xu

**Affiliations:** ^1^ Fujian Key Laboratory of Prenatal Diagnosis and Birth Defect, Medical Genetic Diagnosis and Therapy Center, Fujian Maternity and Child Health Hospital, Fuzhou, Fujian, China; ^2^ Department of Clinical Laboratory, Fuzhou First General Hospital, Fuzhou, Fujian, China; ^3^ Department of Gynecology, Fujian Maternity and Child Health Hospital, College of Clinical Medicine for Obstetrics & Gynecology and Pediatrics, Fujian Medical University, Fuzhou, Fujian, China

**Keywords:** *DMD*, asymptomatic, SNP-array, MLPA, LRS

## Abstract

**Background:**

*DMD* genetic variants cause a spectrum of phenotypes, from severe progressive proximal muscle weakness and degeneration leading to wheelchair dependence and death from cardiac and/or respiratory failure to very mild muscular phenotypes; very rarely, cases are completely asymptomatic. Few cases have been reported in males carrying *DMD* deletions who are asymptomatic.

**Methods:**

Family clinical information was collected from the patients. A single nucleotide polymorphism array (SNP-array) was used to detect abnormalities in prenatal diagnosis, and multiplex ligation-dependent probe amplification (MLPA) and long-read sequencing (LRS) were used to confirm the detected variant.

**Results:**

We incidentally identified *DMD* exons 48–55 deletion using SNP-array in prenatal diagnosis; the variant was confirmed using MLPA and LRS, and the fragment size and precise locations of breakpoints were determined. The variant was precisely located at genomic position chrX:31640088–31945085, spanning from intron 47 to intron 56 in *DMD*. Serum biochemical indicators were normal in the male with the deletion.

**Conclusion:**

Our study is the first to report a *DMD* exons 48–55 deletion in prenatal diagnosis. The phenotypes of *DMD* variants are diverse, and this study suggests that prediction of clinical severity based solely on molecular findings should be interpreted with caution.

## 1 Introduction

Duchenne muscular dystrophy (DMD) is an X-linked recessive disorder characterized by progressive muscle degeneration and weakness caused by variations in the *DMD* gene, including deletions (68%), duplications (10%), small variants such as point mutations (22%), and rare complex rearrangements (<0.5%), which may vary slightly in different regions (1–3). It usually occurs in males and affects 1/6,000–1/3,600 live births worldwide (4). The clinical phenotypes of muscular dystrophy associated with mutations in *DMD* are variable, ranging from severe DMD and mild Becker's muscular dystrophy (BMD) to an extremely rare, completely asymptomatic phenotype (5–7).

*DMD* encodes dystrophin and includes 79 exons spanning 2.2 Mb on Xp21.1; it is the largest gene in the human genome. Traditional molecular detection methods such as multiplex ligation-dependent probe amplification (MLPA) are widely used for DMD mutation screening in clinical laboratories to rapidly and effectively detect exon copy number variations. Next-generation sequencing (NGS) technology enables the detection of single nucleotide variations among the 79 exons within *DMD*. MLPA with NGS is commonly used clinically to screen for *DMD* abnormalities, and more than 90% of the variations can be detected in patients with DMD/BMD (8, 9). However, traditional methods cannot identify complex structural variants or determine whether the variations occur intragenically or extragenically. Long-read sequencing (LRS) can effectively discriminate the precise physical locations of breakpoints and structural features of genomic rearrangement and is increasingly being adopted (10–14). With newly emerging genetic technologies, more *DMD* variations unrelated to indications are being detected.

Here, we report a *DMD* exons 48–55 deletion in clinical that was inadvertently detected during prenatal diagnosis using a single nucleotide polymorphism array (SNP-array) and confirmed with MLPA and LRS. Our study reinforces the importance of detailed clinical evaluation, precise molecular testing, and appropriate genetic counseling when clinical phenotypes are inconsistent with genotypes.

## 2 Materials and methods

### 2.1 Participants

A 35-year-old pregnant female was admitted to our hospital for prenatal diagnosis at an advanced gestational age of 18 weeks. She had no family history of complications. Her spouse was 35 years old and showed no symptoms related to DMD or BMD (such as myasthenia, amyotrophia, pseudohypertrophy of the gastrocnemius muscle); in addition, he reported no muscle fatigue, or walking discomfort. Although he refused to undergo muscle testing, a physical examination showed that, his upper and lower limbs demonstrated sequential joint extension and flexion movements, and could resist force exerted by the examiner, showing normal muscle strength. He did not have cardiac discomfort and also refused to undergo cardiac-related examinations (echocardiography etc.), and nor a family history of muscle or heart diseases. The biochemical indicators showed normal serum creatine kinase (CK) (198 IU/L, reference range: 20–270 IU/L), creatine kinase isoenzyme MB (CK-MB) (18 IU/L, reference range: 0–32 IU/L), alanine aminotransferase (ALT) (27.0 IU/L, reference range: 7–50 IU/L), aspartate aminotransferase (AST) (22.6 IU/L, reference range: 12–40 IU/L), lactate dehydrogenase (LDH) (166 IU/L, reference range: 90–282 IU/L), and hypersensitive troponin Ⅰ (TNIU) (<0.01 μg/L, reference range: 0–0.01 μg/L) concentrations. Informed consent was obtained for all study subjects prior to the molecular studies. This study was approved by the Ethical Review Committee of Fujian Maternity and Child Health Hospital.

### 2.2 Sample collection

Amniocentesis was performed in pregnant women under ultrasound guidance, and 25–30 ml of amniotic fluid was sampled for cell culture, SNP-array, MLPA, and LRS. Peripheral blood was drawn by venipuncture with 5% ethylene-diamine tetraacetic acid as anticoagulant for the pregnant female and her spouse, the other pedigree members were not genetically tested. Genomic deoxyribonucleic acid (DNA) was extracted using the QIAGEN DNA Mini Kit (Qiagen, Hilden, Germany) following standard procedures, the extracted DNA was quantified by a Nano Drop 2000 Spectrophotometer (Thermo Fisher Scientifc, MA, USA) to ensure DNA concentration was >50 ng/μl, and the 260/280 nm optical density ratio was 1.8–2.0.

### 2.3 Single nucleotide polymorphism array

Chromosomal microarray analysis was performed using the CytoScan 750K array with 550,000 nonpolymorphic probes for determination of copy number changes and 200,000 single nucleotide polymorphism probes for allelic confirmation of copy number changes and detection of loss of heterozygosity. Array analyses were performed using the Chromosome Analysis Suite software (ChAS), version 3.3 (Affymetrix, Santa Clara, CA, USA). CNVs analysis was performed using the DGV (http://dgv.tcag.ca/dgv/app/home), DECIPHER (https://decipher.sanger.ac.uk/), ClinVar (https://www.ncbi.nlm.nih.gov/clinvar/), ClinGen Dosage Sensitivity Map (https://dosage.clinicalgenome.org), OMIM (https://omim.org/) for analysis of genes associated with diseases.

### 2.4 Multiplex ligation-dependent probe amplification

MLPA was performed to detect the exonic deletions and duplications of DMD using the SALSA P034/P035 DMD Kit (MRC Holland, Amsterdam, the Netherlands) that according to the manufacturer's recommendations. ABI 3500xL (Applied Biosystems, USA) was used to detect the reaction products. Data analysis was performed using Coffalyser (MRC Holland, Amsterdam, the Netherlands) software. The reference samples were tested simultaneously with clinical samples.

### 2.5 Long-read sequencing

Genomic DNA was sequenced using PacBio SMRT target sequencing of the whole *DMD* (Grandomics, Beijing, China) according to the standard manufacturer's conditions. The Qubit 3.0 (Thermo Fisher Scientific Inc, Carlsbad, CA, USA) and agarose gel electrophoresis was assessed of DNA quantity and quality, respectively. Purified DNA fragments were amplified by polymerase chain reaction and quantified, then subjected to sequencing on the Pacific Biosciences with Sequel II platform according to the manufacturer standard protocols. The data were read and analyzed by bioinformatics after the sequencing data were assessed as qualified by SMRT Link.

### 2.6 Follow-up of pregnancy outcomes

The pregnant outcomes and the growth and development of the baby were collected through review of medical records and telephone follow-up.

### 2.7 Ethics approval and consent to participate

All procedures were performed in accordance with the ethical standards laid down in the 1964 Declaration of Helsinki and its later amendments. Written informed consent to participate was obtained from all the participants, including the minors that were signed by their parent. This research received approval from the Ethical Review Committee of Fujian Maternity and Child Health Hospital (Approval Number: 2023KYLLR01016).

## 3 Results

### 3.1 SNP-array

**Figure 1:**
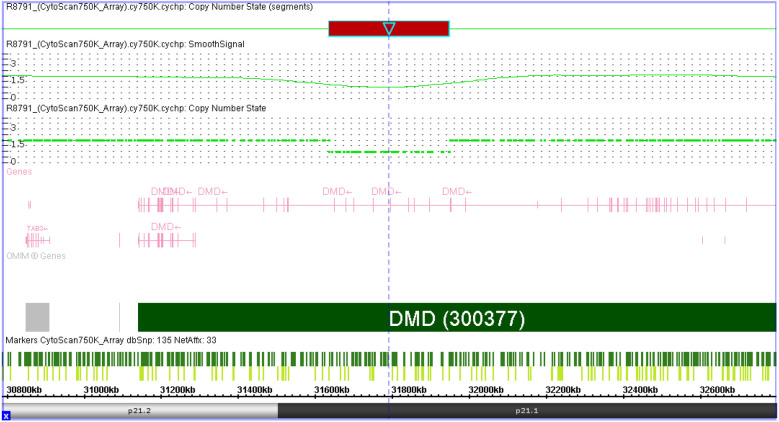
Result of SNP-array detected in female fetus. SNP-array result showed arr[hg19] Xp21.1(31632790_31944075) x1, suggesting a deletion spanning approximately 311 kb within exons 48–55 of *DMD*.

The SNP array on amniocytes showed arr[hg19] Xp21.1(31632790_31944075) x1 in the female fetus, suggesting a deletion spanning approximately 311 kb within exons 48–55 of *DMD* (NM_004006.3). The results of the SNP-array in the parents confirmed that the deletion was derived from the father. No mutations were detected in the mother (Figure 1).

### 3.2 MLPA results

**Figure 2:**
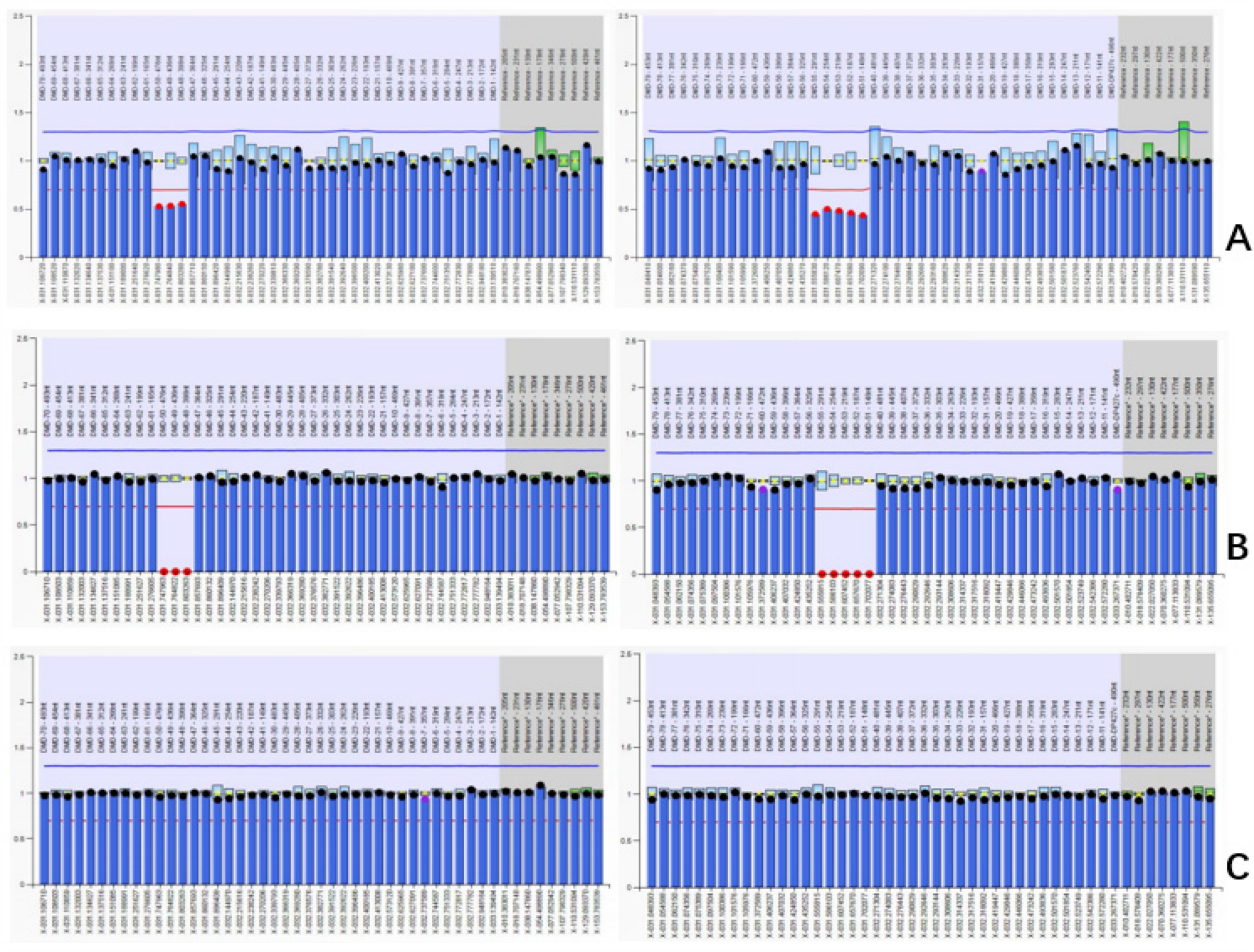
Results of MLPA in the family **(A)** *DMD* exon 48–55 heterozygous deletion in female fetus. **(B)** *DMD* exon 48–55 hemizygous deletion in father. **(C)** Normal copy number in mother.

In the fetus and her father, the deletion variant of exons 48–55 in *DMD* was confirmed, and no deletion/duplication variants were detected in the mother (Figure 2).

### 3.3 Breakpoint identification by LRS

**Figure 3:**
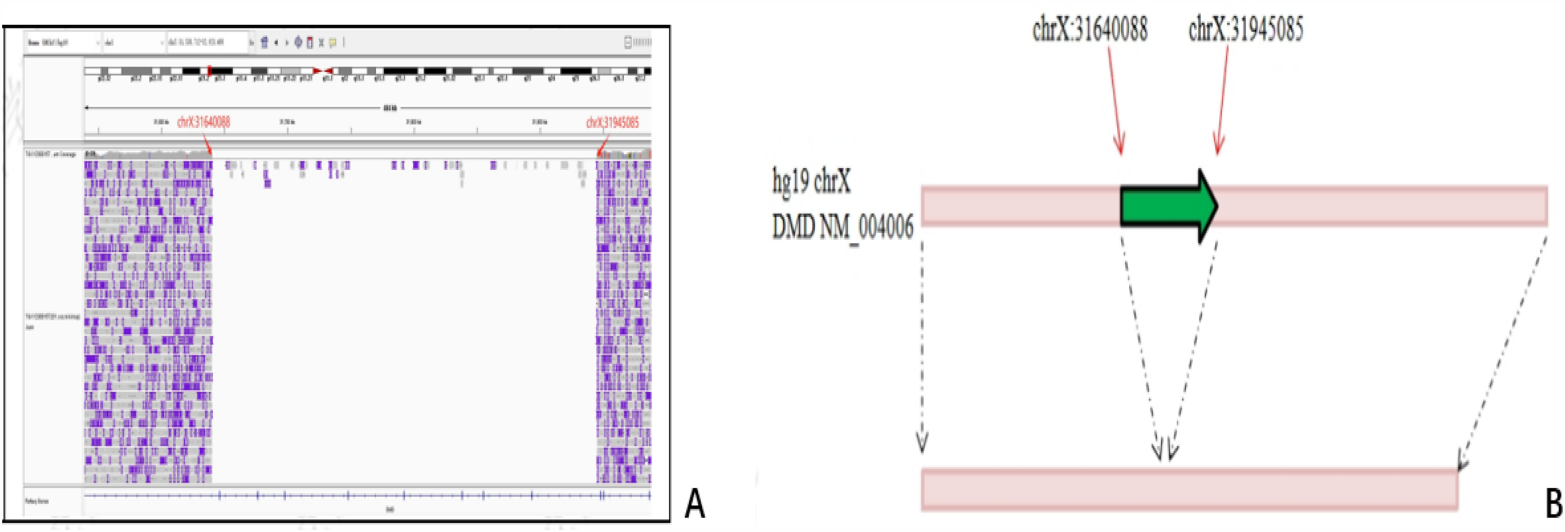
Breakpoint identification by LRS in fetus and her father. A 305 kb deletion spanning chrXp21.1 was identified, and the breakpoints were precisely located at genomic chrX:31640088–31945085, located within introns 47 and 56 in *DMD*. **(A)** Diagrammatic of Binary Alignment/Map format. **(B)** Schematic representation of variation. Red arrows: the breakpoints; Green arrows: the deleted region.

A 305 kb deletion spanning chrXp21.1 was identified in the father and fetus, and the breakpoints were precisely located at genomic chrX:31640088–31945085, located within introns 47 and 56 in *DMD* (Figure 3).

### 3.4 Follow-up

The female vaginally delivered a full-term female baby, and no abnormalities were observed in the baby at birth. The baby was followed up until 1 year of age, and her growth and development were normal.

## 4 Discussion

In clinical practice, various methods have been used to diagnose mutations in patients with DMD and BMD. Variations in *DMD* are mainly caused by deletions or duplications. MLPA is the primary method used to detect copy number variations within the 79 exons of this gene, and approximately 78% of patients can be diagnosed with this test (1). Many laboratories combine MLPA and NGS to screen for *DMD* abnormalities, allowing more than 90% of variations to be detected (8, 9). However, even after using these techniques, a few patients remain genetically undiagnosed owing to the presence of rare deep intronic and complex structural variants. LRS is increasingly being adopted to solve these difficulties in clinical settings and may provide a more effective method for discriminating the precise locations of breakpoints and structural features of genomic rearrangement through the entire *DMD* sequence (10–14).

In this study, we identified a deletion using SNP-array in amniocytes that showed arr[hg19] Xp21.1(31632790_31944075) x1 in the female fetus, suggesting a deletion spanning approximately 311 kb within exons 48–55 in *DMD* (NM_004006.3). The results of the parental SNP-array confirmed that the deletion of *DMD* was derived from the father; this was subsequently confirmed using MLPA, which identified a *DMD* exons 48–55 heterozygous deletion in the female fetus and hemizygous deletion in the father. A deletion of *DMD* exons 48–55 and flanking regions, which were located within intron 47 to intron 55 of *DMD* spanning 305 kb in chrXp21.1 with a precise position in chrX:31640088–31945085, was identified through LRS.

Due to the X-linked inheritance of dystrophinopathies, the majority of female carriers with pathogenic *DMD* variants have no clinical manifestations, but 2.5%–25.7% have varying degrees of skeletal muscle and/or myocardial involvement (15, 16). Given the normal values of biochemical indicators and the lack of symptoms related to DMD/BMD in the father, we suspected that the *DMD* deletions (exons 48–55) in the family were benign variants, and it was recommended that the fetus be preserved. The baby was born without complications and was followed up until 1 year of age, and her growth and development were normal.

Many *DMD* mutations have been identified that produce non- or partially functional dystrophins, which encompass a broad range of clinical manifestations. Severe cases may manifest as a progressive muscle wasting disease that leads to wheelchair dependence at approximately 10 years of age and premature death with cardiac and/or respiratory failure at approximately 20 years of age without treatment; milder cases can have a later onset and slower progression of independent ambulation or may even be asymptomatic (5–7, 17, 18). Phenotypic differences in DMD and BMD are mainly predicted using the reading frame. Mutations that disrupt the reading frame (out-of-frame) produce no or minimal amounts of dystrophin, which results in a DMD phenotype, whereas mutations that leave the reading frame intact (in-frame) typically result in the production of truncated but partially functional dystrophin that is milder and manifests as BMD. Approximately 90% of cases are in agreement with this rule; however, a fraction is not (19–22). With the development of molecular technologies, more mild and asymptomatic cases have been reported. Some asymptomatic cases with *DMD* mutations have complex structural variants, comprising a complete copy as well as a recombination fragment of *DMD*, or are external variations of *DMD*, thus preserving normal *DMD* function (10, 23, 24). However, the attenuating factors in many mild and asymptomatic cases of dystrophinopathies have remained unclear. Changes in the intronic architecture at the variant junctions may result in altered splicing signals, that promote aberrant splicing events; despite the relative inefficiency of altered splicing, asymptomatic and mild BMD phenotypes might result from the presence of residual levels of wild type dystrophin transcripts produced by normal splicing, maintaining full or partial dystrophin function (25–27). Some *DMD* mutations alter splicing and translation sites, resulting in partial exon skipping and bypassing the pathogenic variant; the resulting production of internally shortened but functional proteins may cause a milder phenotype (28–31). Phenotypes might also be influenced by genetic modifiers; for example, *LTBP4* has been reported as a modifier of ambulation, and *THBS1* plays a role in phenotype mitigating (32). More studies are needed to research other factors that influence clinical phenotypes.

Our results showed the deletion of *DMD* exons 48–55, which was predicted to be in-frame by the reading frame rule. However, the male had no symptoms related to DMD or BMD, such as myasthenia, amyotrophia, pseudohypertrophy of the gastrocnemius muscle, or cardiac discomfort, the physical examinations of the asymptomatic male showed normal muscle strength, and his biochemical indicators were normal. To the best of our knowledge, this is the first report of a likely asymptomatic male patient with a deletion of *DMD* exons 48–55. The region between exons 45–55 was a frequent and known “hotspot” for *DMD* mutations, and the deletion of *DMD* exons 48–55 has been reported in some studies (33–35). Diegoli et al. (27) reported a male patient diagnosed with DCM with *DMD* exons 48–55 deletion; his serum creatine phosphokinase level was 456 mU/ml (normal: <200 mU/ml), and an electrocardiogram displayed a left bundle branch block. Baseline echocardiographic results showed that the left ventricular ejection fraction was 20%, and the left ventricular end-diastolic diameter was 78 mm, with a New York Heart Association functional class Functional Class III. The patient eventually died of congestive heart failure. De Palma et al. (28) reported two patients with DMD/BMD due to the deletion of *DMD* exons 48–55 in a pilot study in a southern Italian cohort, but the related clinical manifestations were not described. In a study by Luce et al. (29), a mother and child were identified as asymptomatic females with *DMD* exons 48–55 deletion. In our study, the asymptomatic male patient was 35 years old; whether related symptoms would appear with age requires further follow-up.

*DMD* is one of the largest known genes in humans and encodes a 427 kDa dystrophin protein. Full-length dystrophins are composed of four major domains: an N-terminal actin F-binding domain (encoded by exons 1–8), a large central rod domain (encoded by exons 8–64), a cysteine-rich region (encoded by exons 64–70), and a C-terminal domain (encoded by exons 71–79) (36, 37). Dystrophin is essential as a shock absorber in muscle fiber contraction, physically anchoring cellular skeletal actin fibers and mediating interactions between the cytoskeleton, membrane, and extracellular matrix (38–40). Four dystrophin hinges in the rod domain influence muscle maturation and maintenance. The hinge 3 region is particularly prone to deletion mutations, which are expected to lead to BMD with great heterogeneity in clinical manifestations (18, 41). We report an asymptomatic pedigree of deleted exons 48–55 in *DMD*, in which the deletion region included hinge 3. The frequency of this variant in the Database of Genomic Variants and gnomADSV general East Asian population databases was zero. It is exceptionally rare for male *DMD* patients with dystrophin mutations to remain asymptomatic throughout life. Although the male patient in our study was asymptomatic, regular muscle and cardiac evaluations would be recommended to identify the relevant clinical features that may progress with age.

In conclusion, prenatal diagnosis with no family history of DMD/BMD may incidentally detect *DMD* mutations in fetuses. Our study highlights the importance of detailed clinical evaluations and accurate molecular detection. Precise breakpoint analysis is necessary when clinical manifestations are inconsistent with genotype, and the combined application of multiple techniques can be more comprehensive in evaluating pathogenic differences. When variants are incidental findings in a prenatal diagnosis, caution should be exercised to provide accurate guidance for genetic counseling and prenatal diagnosis.

## Data Availability

The original contributions presented in the study are publicly available. These data can be found here: [ClinVar/accession number SUB15209272 and SUB15243222].
